# Long-term evolution of hydrographic network density in arid Xinjiang, China: reconstruction from historical maps (1900–2020)

**DOI:** 10.1038/s41598-026-49759-7

**Published:** 2026-04-22

**Authors:** Raorao Su, Zhen Zhao

**Affiliations:** 1 School of Economics and Statistics, Guangzhou University, Guangzhou, 510000 China; 2 School of Geography and Remote Sensing, Guangzhou University, Guangzhou, 510000 China; 3 School of History, Renmin University of China, Beijing, 100872 China; 4 Research Institute of Global Chinese and Area Studies, Huaqiao University, Xiamen, 361021 China

**Keywords:** Climate sciences, Environmental sciences, Hydrology

## Abstract

Water security in arid regions has become a critical challenge under ongoing global environmental change. Using arid Xinjiang as a case study, this research reconstructs the spatiotemporal evolution of hydrographic network density (HND) over the past 120 years (1900–2020s) based on historical maps and GIS-based spatial analysis. A grid-based HND dataset was developed for five representative periods to examine long-term structural changes in regional water systems. The results reveal a clear transition from early spatial dispersion to post-mid-20th-century aggregation and subsequent stabilization, with the 1960s marking a key turning point in hydrographic network organization. Spatial autocorrelation increased steadily over time, indicating the emergence of increasingly structured and clustered water systems. Climatic aridity factors–particularly the Palmer Drought Severity Index (PDSI)–exerted dominant controls on hydrographic dynamics in mountainous regions, whereas changes in oasis–plain systems were increasingly driven by human activities, including cropland expansion, irrigation development, and policy-led water management since the mid-20th century. By integrating long-term historical cartographic data with quantitative spatial analysis, this study provides new empirical evidence on how climate forcing and human engineering have jointly shaped water-system evolution in arid environments. The findings offer a long-term perspective for understanding human–environment interactions and for informing water-resource management and adaptation strategies in arid regions under future climate change.

## Introduction

An estimated 1–2 billion people worldwide currently face water scarcity, primarily in arid regions^1^. Xinjiang, with its typical arid climate and unique geographical conditions, has long been regarded as an important sample region for studies of arid environments. The river–lake systems in arid regions are not only major carriers of surface water but also fundamental to human survival, forming the basis of the regional water environment–or the broader water ecological system. Existing research has examined the spatial characteristics and evolution of rivers in arid regions from multiple perspectives, such as analyses of the ecological impacts of hydropower development along the Snake River in the western United States^2,3^, as well as studies on the evolution of river network morphology and hydrological regimes in the Tarim River Basin under the combined influences of aeolian processes and human activities in arid Xinjiang^4^. Related work has also investigated historical changes in river systems, including empirical studies on the Tarim, Manas, Shule, and Salawusu rivers, and the Ejin Basin^5–11^.

However, most of these studies are grounded in historical geography and rely primarily on traditional sources such as historical documents, topographic maps, and geomorphological evidence. While these materials provide valuable insights into the evolution of water systems in arid regions, they often suffer from limitations in spatial precision and temporal comparability. In recent years, advances in GIS technology have offered new paradigms and tools for spatiotemporal analysis of historical water systems^5,12,13^. Yet, compared with eastern China’s monsoon regions, GIS-based studies of arid northwestern China remain largely concentrated on the last six decades, a period for which remote sensing data are readily available. Research on longer timescales–extending over a century or more–remains noticeably insufficient. Moreover, existing studies tend to focus on individual river basins or specific river reaches, lacking a systematic understanding of water-network evolution at the broader regional or basin scale.

The long-term evolution of surface water networks is of fundamental multidisciplinary significance, particularly in arid regions where hydrological systems are strongly shaped by climatic variability and human intervention. Long-term climate changes, including sustained warming and intensifying drought, exert profound influences on river–lake dynamics and water availability^14,15^. In oasis-based arid environments, water resources form the critical foundation for both ecological functioning and social persistence. Over extended historical periods, human societies have constructed extensive irrigation infrastructures through artificial canals, producing complex hydrographic networks in which natural river channels and man-made waterways are closely intertwined. Without a long-term, integrated analytical perspective, the structural characteristics and underlying mechanisms of water-system evolution in arid regions risk being underestimated.

In this context, this study adopts hydrographic network density (HND) as a core indicator to characterize the historical structure of surface water systems in arid Xinjiang. By integrating historical maps with GIS-based spatial analysis, we reconstruct a multi-period dataset of water-network density spanning from 1900 to 2020 and systematically examine its spatiotemporal evolution and associated climate–society interactions. As an established geographic metric, water-network density has been widely applied to investigate natural–human relationships, settlement dynamics, and economic processes, demonstrating strong integrative explanatory power. By centering on this indicator at a regional scale and over a long temporal horizon, this study addresses key limitations in existing research concerning temporal depth, spatial coverage, and methodological integration, and provides empirical insights into historical human–environment relationships and contemporary water-security challenges in arid landscapes.

## Study area and methods

### Study area

**Fig. 1:**
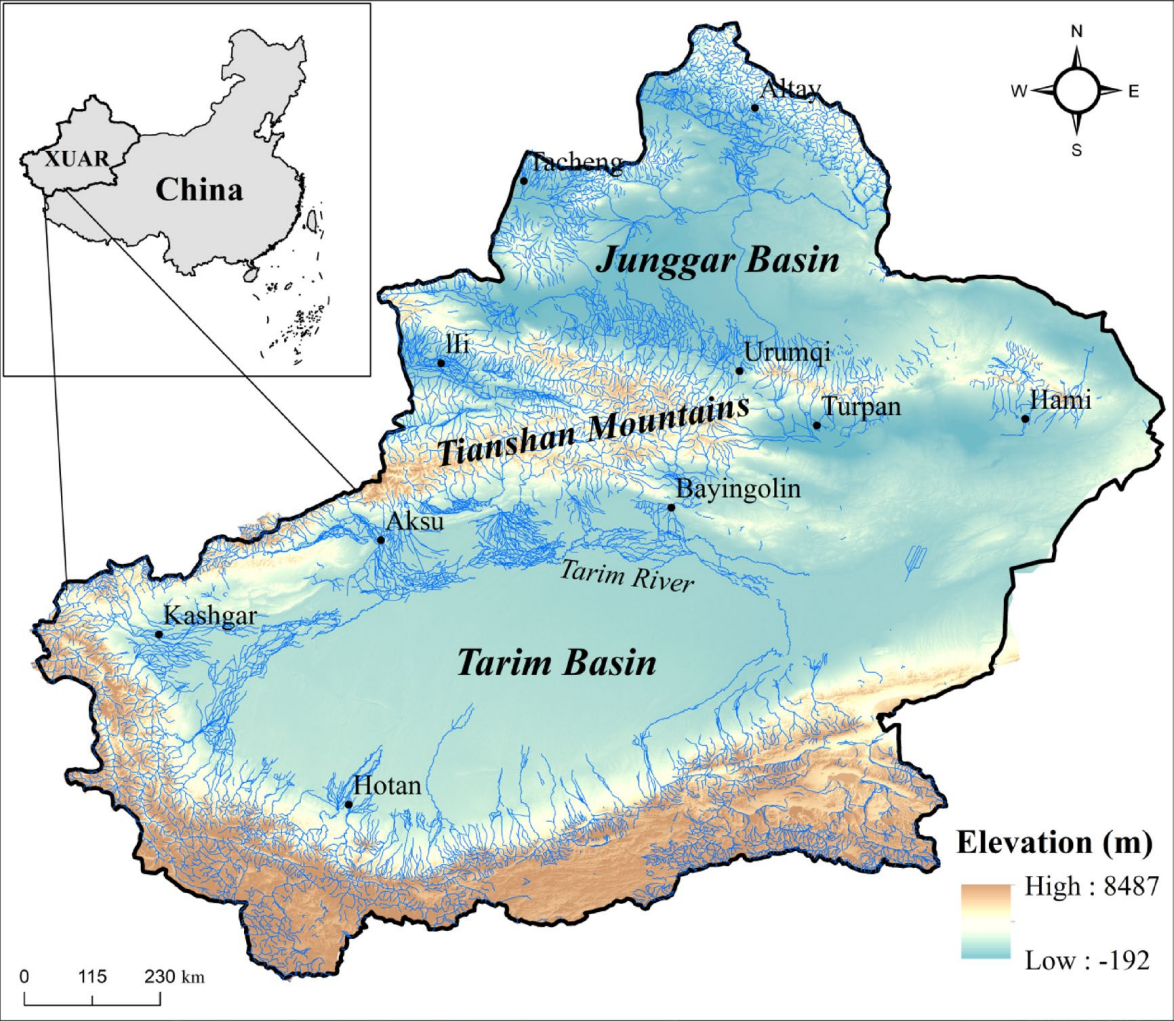
Location of the study area and geomorphological features. Map was created by the authors using ArcGIS 10.8.2 (Esri Inc., Redlands, CA, USA; https://www.esri.com). Elevation data derived from the SRTM DEM (90 m resolution; NASA/USGS; accessed via Geospatial Data Cloud, Computer Network Information Center, Chinese Academy of Sciences, https://www.gscloud.cn/sources/accessdata/305?pid=302). River network and administrative boundaries derived from the 1:1,000,000 Public Edition of Fundamental Geographic Information Data (National Catalogue Service for Geographic Information, https://www.webmap.cn/commres.do?method=result100W). This base map is also used in Figs. 3, 4 and 5. The boundary line of Xinjiang is drawn with reference to the standard map (Approval No. GS (2023) 2767) from the Standard Map Service of the Ministry of Natural Resources of China (http://bzdt.ch.mnr.gov.cn).

Xinjiang, officially designated as the Xinjiang Uygur Autonomous Region (XUAR), lies in northwestern China at the center of the Eurasian continent and represents a typical arid to semi-arid environment (Fig. 1). The region spans 73°40′–96°23′E and 34°25′–49°10′N, covering approximately 1.66 million km². Its physical geography is defined by three major mountain systems–the Altai, Tianshan, and Kunlun ranges–with the Junggar Basin to the north and the Tarim Basin to the south situated between these mountains^16,17^. The Tianshan Mountains divide Xinjiang into Northern and Southern Xinjiang. Northern Xinjiang features a continental arid to semi-arid climate, with mean winter and summer temperatures of − 13 °C and 22.2 °C, respectively; Southern Xinjiang has a continental dry climate, with corresponding averages of − 5.7 °C and 24.4 °C. Annual precipitation is around 210 mm in the north but falls below 100 mm in the south. Owing to extremely dry conditions, evaporation is intense across the region, with annual pan evaporation ranging from 1000 to 4500 mm^16^.

As a typical arid landscape, water is the primary factor sustaining both ecological systems and human societies. Xinjiang is also a key crossroads along the Eurasian Silk Road. Amid vast deserts and gravel plains, numerous oases depend on meltwater from surrounding mountains (Fig. 1). For centuries, oasis communities have constructed irrigation canals and diverted water to support settlement and agriculture, creating a dense and interwoven network of waterways and human habitation. Human activities, particularly large-scale water infrastructure development, have profoundly shaped the hydrographic structure of Xinjiang. Since the mid-20th century, extensive hydraulic engineering projects have been constructed across the region, including diversion headworks, main and lateral irrigation canals, and reservoirs designed to regulate seasonal runoff. For example, the Shengli Reservoir in the Aksu region, with a designed storage capacity of 108 million m³, represents one of many such facilities that have fundamentally altered local water distribution patterns and channel morphology^18^. These large-scale water-management projects increasingly constrained natural channel migration, contributing to a more stabilized but heavily engineered hydrographic structure across oasis–plain areas^19^.

The hydrographic system of Xinjiang is dominated by inland drainage networks, encompassing the Tarim Basin, Junggar Basin, and Ili Valley systems, among others. Regional rivers are characterized by a predominance of small and medium-sized streams, with rivers having an annual runoff of less than 100 million m³ accounting for 85.4% of the total number of rivers in the region. Streamflow is primarily generated in mountainous areas through snowmelt and glacial meltwater, and rivers generally carry high sediment loads. Among the larger rivers with annual runoff exceeding 1 billion m³ are the Ili, Irtysh, Yarkant, Aksu, Kashgar, Hotan, Kaidu, Weigan, Manas, and Ulungur rivers, while the Tarim River and Kongque River constitute the two major trunk rivers of the region^20^. This distinctive hydrographic configuration—where mountain-fed rivers sustain dispersed oasis settlements across vast desert basins—forms the fundamental context for understanding long-term water network evolution in arid Xinjiang.

## Materials and methods

### Map sources and data processing

This study reconstructs a high-resolution hydrographic dataset for Xinjiang over the past 120 years by manually digitizing historical maps and integrating modern GIS data. Hydrographic line features for the 1900s, 1930s, 1960s, and 1990s were extracted through systematic interpretation of historical map legends and visual inspection, and combined with the 1:1,000,000 hydrographic dataset from the National Catalogue Service for Geographic Information (Table 1).

**Fig. 2:**
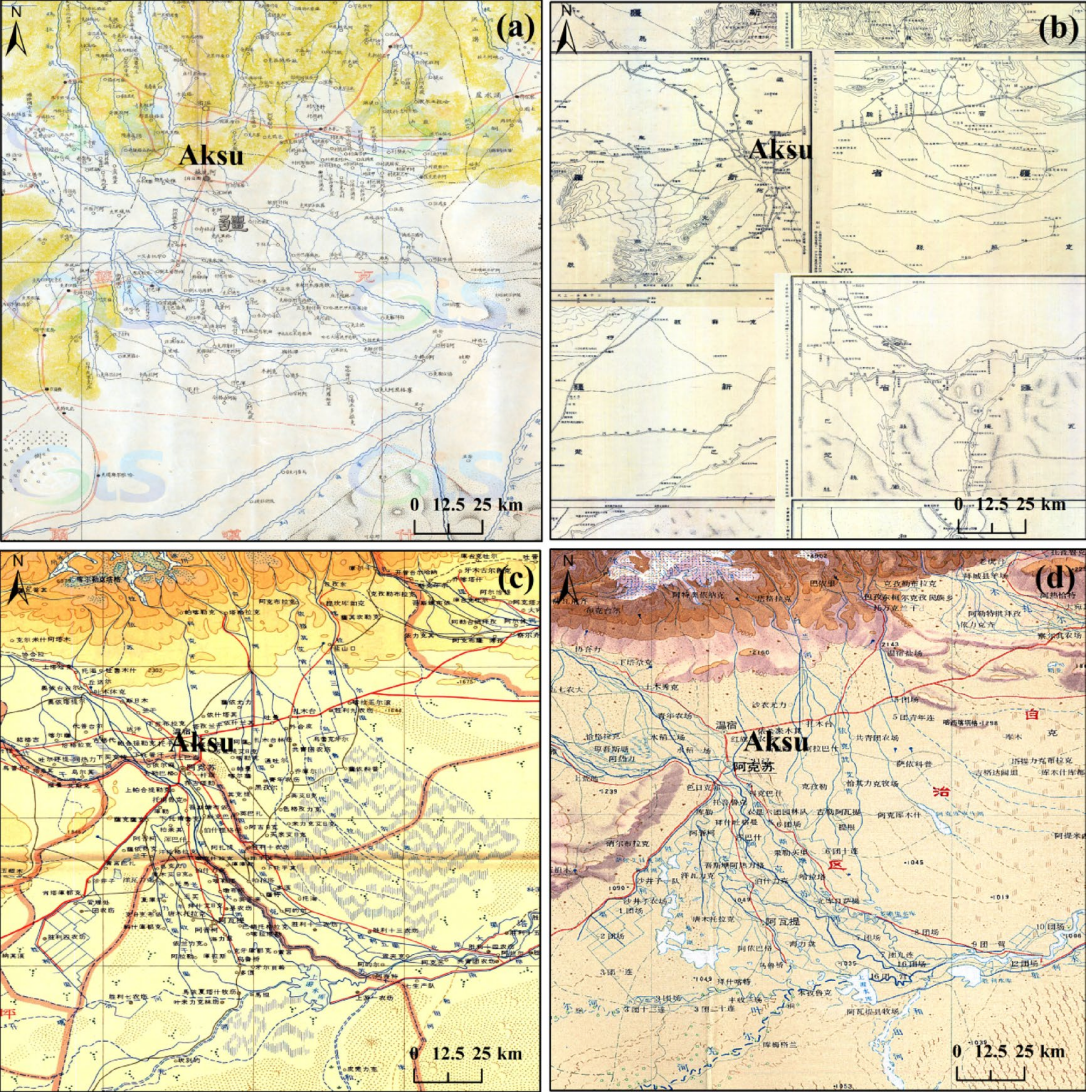
Sample map extracts from each historical source for the Aksu region, illustrating cartographic styles, symbol conventions, and levels of hydrographic detail across the four historical periods: (**a**) 1900s (1:500,000 Chinese Topographic Map, 1917–1918, General Staff Cartography Bureau); (**b**) 1930s (1:300,000 Topographic Map, 1935, Land Survey Bureau of General Staff); (**c**) 1960s (Xinjiang Uygur Autonomous Region Atlas, 1966, Xinjiang Atlas Compilation Committee); (**d**) 1990s (1:1,000,000 Atlas of China, 1997, State Bureau of Surveying and Mapping, SinoMaps Press). Map layout created using ArcGIS 10.8.2 (Esri Inc., Redlands, CA, USA).

Historical maps, despite limitations in geometric accuracy, provide consistent symbolic representations of water features, administrative boundaries, and engineered hydraulic systems. To ensure temporal comparability, watercourse elements explicitly marked on these maps were used as the primary data source, and a standardized workflow was developed for mosaicking, georeferencing, interpretation, and manual vectorization. Cartographic documentation confirms the systematic representation of hydrographic features across all map sources. For the 1900s maps, compilation notes state that the maps were compiled from various sketch maps and supplemented by geographic survey records (Table 1), with minor watercourses including lateral irrigation canals explicitly identified at the map-sheet level. For the 1960s atlas, the preface explicitly states that topographic maps of various scales were used as primary source material, with data collected from county-level units and relevant professional agencies to supplement and verify content (Table 1); the map legend further includes dedicated symbols for reservoirs and irrigation canals. For the 2020s dataset, hydrographic features were extracted from the 1:1,000,000 Public Edition of Fundamental Geographic Information Data (National Catalogue Service for Geographic Information, https://www.webmap.cn/commres.do?method=result100W) following the classification standards of GB/T 13923−2022 (Classification and Codes for Fundamental Geographic Information Feature), including permanent surface rivers (code 210101), intermittent rivers (code 210200), main irrigation canals (code 220200), and branch irrigation canals (code 220300)^21^.The 1930s and 1990s map series similarly include map legends and textual annotations identifying named watercourses (Fig. 2), providing consistent cartographic conventions for hydrographic feature identification^22^.

Because traditional “river network” classifications cannot adequately capture the mixed hydrological landscape of arid Xinjiang–where natural rivers, irrigation canals, ephemeral channels, and drainage ditches frequently overlap–this study uses the broader term “hydrographic system.” A unified hydrographic network density (HND) indicator was constructed to better represent both natural hydrological processes and human-modified water systems.

Historical hydrographic data were derived from four categories of maps and supplementary geographic sources (Table 1). Although map scales vary (1:300,000–1:1,000,000), scale-induced discrepancies were minimized by aggregating all hydrographic features into a uniform 10 km × 10 km grid. This grid-based method emphasizes relative spatial patterns–such as density differences and clustering–within each period, which remain stable across scales and support temporal comparison.

The positional offset of historical maps can be treated as non-systematic error, as discussed in the section on methodological uncertainty. The mean offset distance across all maps is 8.64 km, while the average offset values for the 1900s, 1930s, 1960s, and 1990s are 19.70 km, 11.47 km, 1.84 km, and 1.56 km, respectively. Spatial comparisons between test points and corresponding control points indicate no consistent directional bias, with offset vectors distributed irregularly across different orientations and locations. This pattern suggests that map offset errors are not spatially structured and therefore do not introduce systematic distortion into the grid-based spatial analysis.

All historical maps were mosaicked, georeferenced, interpreted, and vectorized following unified standards. In total, 21,515 hydrographic features were extracted. Map symbols, textual annotations, and cartographic conventions were strictly followed to ensure semantic fidelity and preservation of spatial topology. Given the inherent temporal ambiguity of historical cartographic sources, each map was assigned to its representative decade rather than a specific year, based on documentary evidence including prefaces, compilation records, and dataset metadata (Table 1). This decade-based approach is consistent with standard practice in historical geographic research using multi-source cartographic data.

**Table 1:** List of map data sources and platforms.

Edition/publication year	Reference period	Map name	Scale	Source	Type	Projection / coordinate system
1917–1918	1900s (1900–1910)	1:500,000 Chinese Topographic Map	1:500,000	General Staff Cartography Bureau^a^	Military map	Unknown
1935	1930s (1930–1940)	1:300,000 Topographic Map	1:300,000	Land Survey Bureau of General Staff ^b^	Military map	Unknown
1966	1960s (1960–1970)	Xinjiang Uygur Autonomous Region Atlas	1:750,000, 1:500,000, 1:1,000,000	Xinjiang Atlas Compilation Committee	Internal atlas	Gauss-Krüger projection, datum unknown
1997	1990s (1990–2000)	1:1,000,000 Atlas of China	1:1,000,000	State Bureau of Surveying and Mapping; SinoMaps Press	National atlas	Lambert conformal conic projection (等角圆锥投影), datum unknown
2021	2020s (2019–2020)	1:1,000,000 Public Edition of Fundamental Geographic Information Data	1:1,000,000	National Catalogue Service for Geographic Information, https://www.webmap.cn/commres.do?method=result100W	Dataset	China Geodetic Coordinate System 2000 (CGCS2000)

a and b. Digitized versions are accessible via Academia Sinica Map Archive (https://map.rchss.sinica.edu.tw). All datasets were reprojected to a unified Albers Conical Equal Area projection (GCS: WGS 1984) for spatial analysis.

### Data analysis

Climate data were obtained from the Climatic Research Unit (CRU) gridded climate datasets of the University of East Anglia (UEA) (https://www.uea.ac.uk/groups-and-centres/climatic-research-unit/data). This dataset is widely used in global climate research and provides high-resolution (0.5°) gridded climate variables— including temperature, precipitation, and drought indices—covering global land areas from 1901 onward. Elevation and slope data were sourced from the SRTM DEM (90 m resolution) provided through the Geospatial Data Cloud (https://www.gscloud.cn/sources/accessdata/305?pid=302)^23^. Population data were primarily derived from *The Population of China: Xinjiang Volume*^24^ and successive editions of the *China Population and Employment Statistical Yearbook*. Cropland data were obtained from the Land Use and Land Cover Change (LUCC) dataset of China (1900–2019) provided by the National Science & Technology Infrastructure (http://www.nesdc.org.cn)^25,26^.

All raster datasets were reprojected to a consistent Albers equal-area conic projection matching the study area’s administrative regions and clipped to the Xinjiang extent using GDAL (Python3). Specifically, raster datasets for potential evapotranspiration (PET), precipitation (PRE), Palmer Drought Severity Index (PDSI), and mean temperature (TMP) were extracted for each year.

To reduce inter-annual variability and highlight long-term trends, annual regional statistics for climatic variables (PDSI, PET, PRE, TMP) and cropland area (pixel sum) were aggregated into decadal means using consecutive 10-year windows (1901–1910, 1911–1920, …, 2011–2020). Population data, which are available only for selected years prior to 1949, were linearly interpolated between documented data points to produce a continuous annual series before decadal aggregation. Data sources and interpolated years are detailed in Appendix A. All decadal variables were then standardized to Z-scores across the full time series to enable direct comparison of relative changes despite differing units and magnitudes.

To systematically reconstruct the spatiotemporal evolution of Xinjiang’s hydrographic network over the past 120 years, we developed a five-period water system dataset (1900–2020) based on historical hydrographic data. Three major analytical approaches were employed:

1. Spatial autocorrelation analysis. Global Moran’s *I* was used to assess the spatial autocorrelation of hydrographic network density in each period and to determine whether the overall spatial pattern tended toward clustering or dispersion. Local spatial autocorrelation (Local Moran’s *I*, LISA) was further applied to identify high–high (HH) clusters, low–low (LL) clusters, and spatial outliers (high–low or low–high), thereby revealing localized clustering patterns and anomalous changes.

A consistent set of 15,901 grid cells was used across all five periods, following the exclusion of boundary cells with an area less than 100 km². For each valid grid cell, HND was calculated as the total watercourse length (km) divided by the cell area (100 km²), and the regional HND for each period was reported as the mean across all valid cells. Grid cells with zero watercourse length were retained as valid observations representing areas without hydrographic features, thereby preserving the true spatial distribution and avoiding bias in spatial autocorrelation results. For both Global Moran’s *I* and Local Moran’s *I* (LISA) analyses, spatial relationships were defined using a rook contiguity weights matrix (shared edges only) with row standardization applied. Statistical significance for LISA was evaluated using 999 random permutations at *p* < 0.05. FDR correction was tested but not applied, as it returned no significant clusters in several periods.

(2) Density change-rate analysis. Using a 10 km × 10 km grid as the basic analytical unit, we calculated the total length of watercourses per grid cell to derive hydrographic network density (km/km²). By comparing densities between consecutive time periods, we generated maps of spatial change rates to identify regions of expansion, contraction, or directional migration of the water network.
(3) Regional heterogeneity analysis. Grid cells were classified into mountainous and oasis–plain zones based on mean elevation extracted using the Zonal Statistics as Table tool (ArcGIS 10.8.2), applied to the SRTM DEM (90 m resolution) within each 10 km × 10 km grid cell. Grid cells with a mean elevation ≥ 1,500 m were assigned to the mountainous zone, while those below this threshold were assigned to the oasis–plain zone. HND values were then compared between zones across all five periods to examine distinct trajectories of hydrological change under different geomorphological and water-management regimes.

In addition, historical literature–including gazetteers, archival records, and documentary materials–was integrated to contextualize key phases of water system change and interpret the socio-institutional and anthropogenic drivers behind these transformations.

Together, these methods form a comprehensive analytical framework that examines hydrographic network evolution from the perspectives of overall spatial structure, spatial autocorrelation, local dynamics, and regional heterogeneity, ensuring both comparability across periods and strong explanatory power for long-term spatiotemporal patterns.

## Results

### Trends in hydrographic network density

From 1900 to 2020, HND exhibited a clear trajectory of dispersed → clustered → spatially balanced development. In the early 20th century, density already aligned with the Tianshan Range and Tarim Basin margins. From the mid-20th century onward, hydrographic networks underwent significant spatial reorganization and became more evenly distributed. By the 2020s, spatial disparities further decreased, suggesting a transition toward a more uniform hydrographic network structure across Xinjiang.

Based on our calculations, the mean HND across all valid grid cells in the five examined periods–1900s, 1930s, 1960s, 1990s, and 2020s–was 0.0547, 0.0571, 0.0556, 0.0704, and 0.0808 km/km², respectively.

**Fig. 3:**
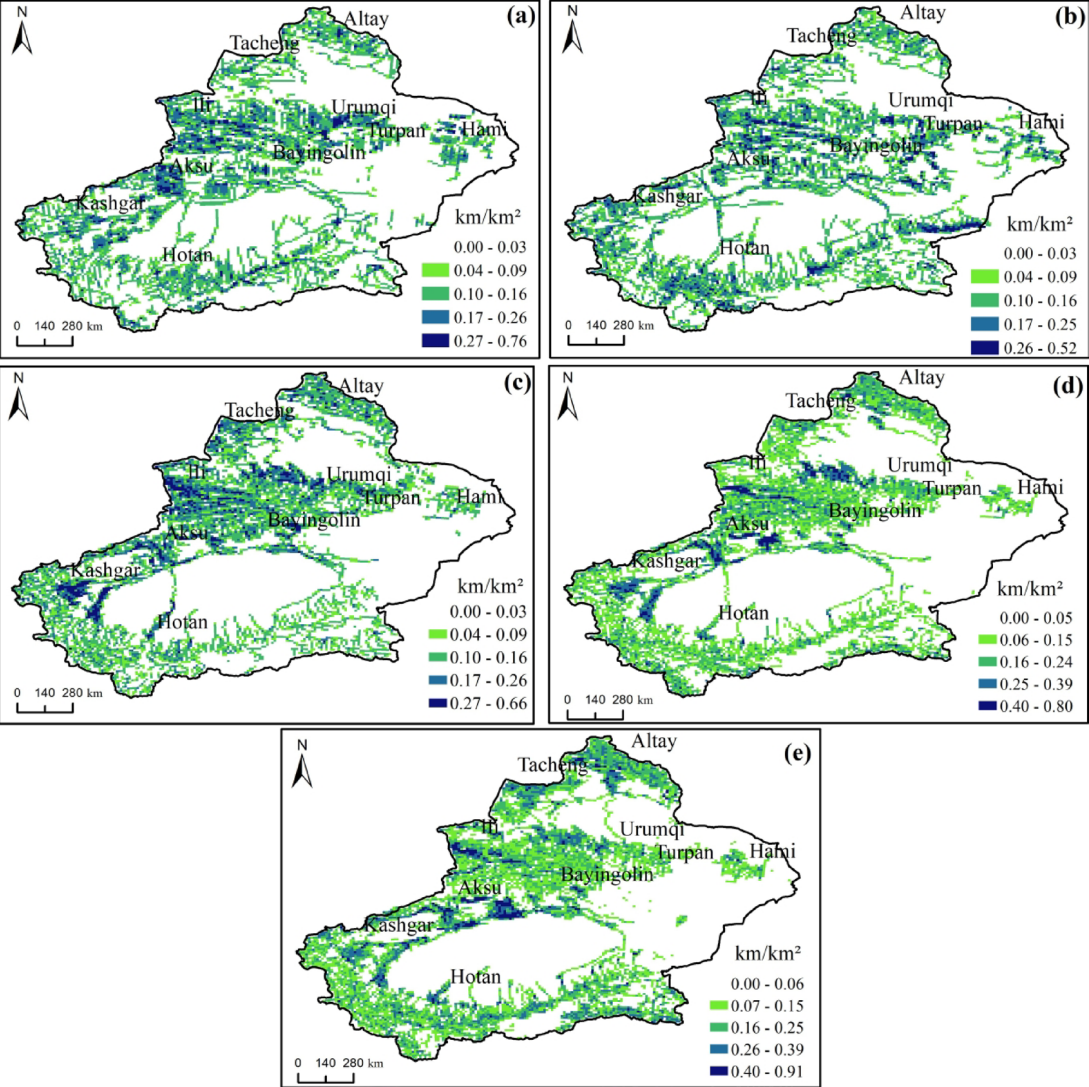
Spatial distribution of hydrographic network density (HND) in Xinjiang during five periods: (**a**) 1900s, (**b**) 1930s, (**c**) 1960s, (**d**) 1990s, and (**e**) 2020s. Maps created by the authors using ArcGIS 10.8.2 (Esri Inc., Redlands, CA, USA).

Figure 3 presents the spatial distribution and temporal evolution of HND in Xinjiang from 1900 to 2020.

1. 1900s (Fig. 3a). HND formed two major concentration belts: a northwest–southeast belt linking Ili to Urumqi, and a southeast–northwest belt from Aksu to Turpan. Together, these created a triangular cluster centered on the Tianshan Range. Additional belt-shaped clusters occurred along the Kashi and Yarkant river oases, the southern margin of the Tarim Basin (Hotan–Qiemo), as well as along the Tarim River and in Hami.
2. 1930s (Fig. 3b). The HND clusters expanded into a rectangular pattern with Ili, Urumqi, Yanqi Basin, and Aksu as four vertices, still oriented along the Tianshan axis. Density values decreased in the Aksu, Yarkant, and Kashi belts, while the high-density ring along the southwestern–southern margin of the Tarim Basin (Kashi–Pishan–Hotan–Qiemo) expanded. Distinct high-density centers began to emerge within the clusters.
3. 1960s (Fig. 3c). Hydrographic networks expanded markedly across Xinjiang in spatial extent and clustering, particularly in oasis–plain areas, although the regional mean HND (0.0556 km/km²) remained marginally below the 1930s level (0.0571 km/km²), reflecting concurrent sharp declines in eastern Xinjiang.
4. 1990s (Fig. 3d). The overall pattern of the 1960s persisted, but the hierarchical structure of HND became more pronounced. High-density oases became more clearly defined in Urumqi–Wusu, Ili Valley, Aksu, Kuqa, Kashi, Yarkant, and Hotan.
5. 2020s (Fig. 3e). While maintaining the high-density regions of the 1990s, spatial differences further diminished. Density expanded in the Junggar Basin and Altay region, while high-density clusters in the lower Hotan and Kongque rivers disappeared.

### Local clustering and changes in spatial aggregation patterns

Table 2 summarizes the global spatial autocorrelation of hydrographic network density in Xinjiang from the 1900s to the 2020s. Moran’s *I* values remain consistently positive across all periods, indicating strong spatial clustering. Specifically, Moran’s *I* increased from 0.56 in the 1900s to 0.67 in the 2020s, with all corresponding Z-scores exceeding 95, demonstrating statistically significant clustering. This upward trend indicates that spatial aggregation of hydrographic density has intensified over the past 120 years, evolving from relatively dispersed to more concentrated patterns.

**Table 2:** Spatial autocorrelation results of hydrographic network density in Xinjiang over the past 120 years.

Period	1900s	1930s	1960s	1990s	2020s
Moran’s *I*	0.56***	0.54***	0.61***	0.67***	0.67***
Z-score	99.43	95.76	107.00	117.50	117.92

*** Indicates statistical significance at *p* < 0.001 level.

**Fig. 4:**
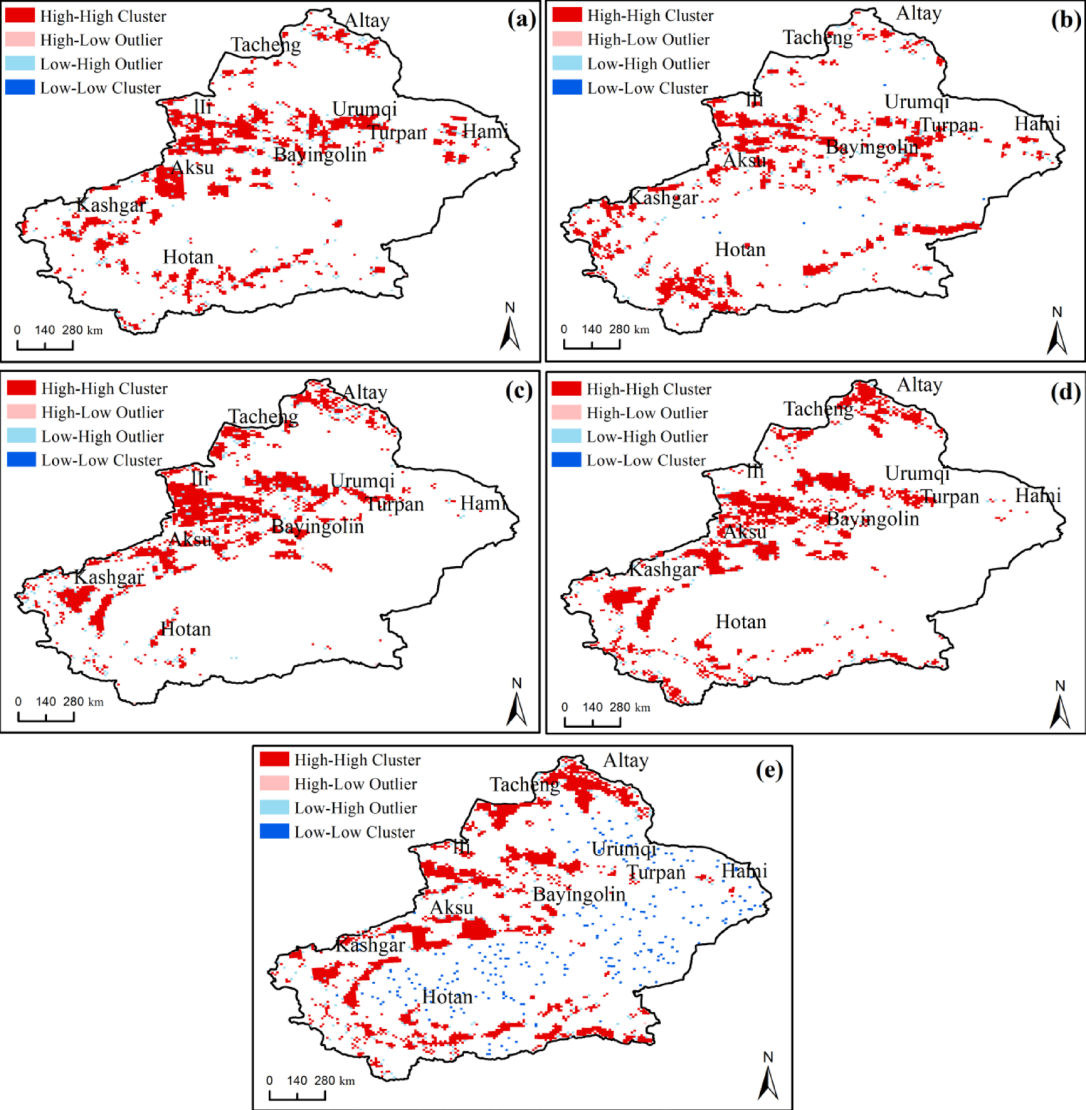
Local Moran’s *I* Distribution Map of HND in Xinjiang: (**a**) 1900s, (**b**) 1930s, (**c**) 1960s, (**d**) 1990s, (**e**) 2020s. Maps created by the authors using ArcGIS 10.8.2 (Esri Inc., Redlands, CA, USA).

Figure 4 illustrates the spatial distribution of Anselin Local Moran’s *I* (LISA) for the five representative periods. Compared with mountain regions, oasis–plain areas exhibit more pronounced and persistent high–high (HH) clusters, characterized by their dense and highly organized hydrographic networks. The HH clusters are mainly concentrated along the northern foothills of the Tianshan Mountains, the Ili Valley, and the southeastern–northwestern and northeastern–southwestern margins of the Tarim Basin. These areas either possess abundant natural water resources or well-developed irrigation infrastructure, functioning as core zones of high HND. Notably, HH clusters diminish markedly during the 1930s, indicating a temporary shift toward spatial dispersion. The 1930s also witnessed the emergence of localized low–low (LL) clusters along the middle and lower reaches of rivers in both the Tarim and Junggar basins, as well as scattered LL patterns within the interior of these basins. This pattern is consistent with the social instability and internal conflicts of this period, which disrupted irrigation maintenance and led to the contraction of water systems in middle- and lower-reach areas that depend heavily on artificial diversion infrastructure. As surrounding oasis HND declined, the contrast between low-density interior zones and adjacent areas diminished, rendering previously non-significant low-value clusters statistically detectable. This interpretation aligns with the documented sharp decline in cultivated land during the 1930s^27^.

In contrast, low–low (LL) clusters primarily appear in regions where extreme climate and geomorphological constraints limit water system development. Although the Tarim Desert remained a zone of very low hydrographic density from 1900 to 1990, it did not form statistically significant clustering due to its internally uniform distribution. Only in the 2020s, as hydrographic density increased substantially in surrounding regions, did the desert area begin to display a statistically significant LL cluster, indicating increasing structural differentiation and the emergence of a “low-value core” within the regional hydrographic system.

Occurrences of low–high (LH) and high–low (HL) outlier patterns are rare across all periods, suggesting that abrupt, localized changes in hydrographic density were uncommon.

### Regional density change rates and identification of expansion and contraction zones

**Fig. 5:**
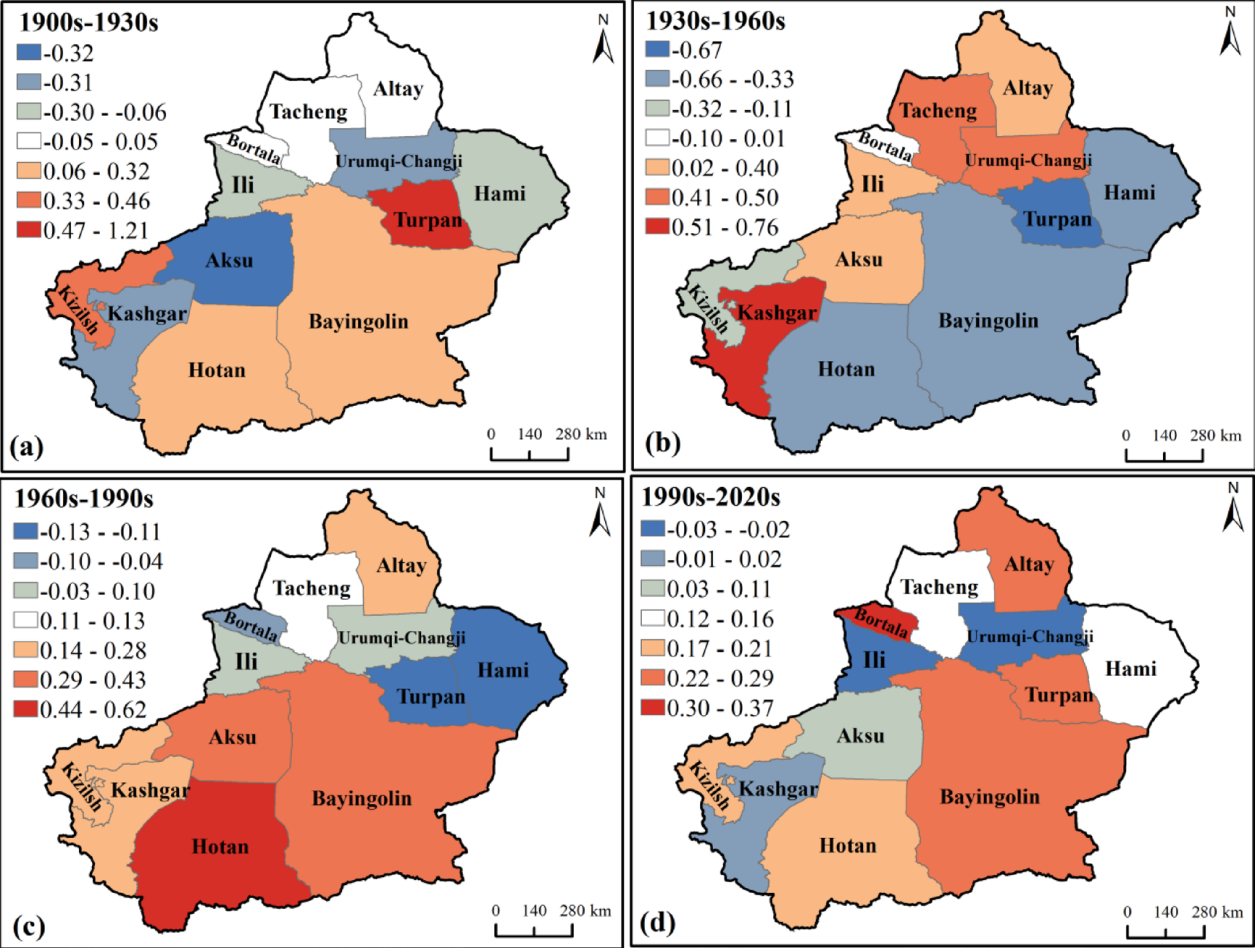
Temporal variation characteristics of HND across regions in Xinjiang (1900–2020s). Panels (**a**–**d**) show HND changes during the periods 1900–1930s, 1930–1960s, 1960–1990s, and 1990–2020s, respectively. The analysis is based on prefecture-level divisions (regions), with Urumqi and Changji merged into a single analytical unit; sub-prefecture administrative entities, including the Xinjiang Production and Construction Corps, are aggregated into their respective prefecture-level units. Map created by the authors using ArcGIS 10.8.2 (Esri Inc., Redlands, CA, USA; https://www.esri.com).

Figure 5 illustrates the relative change rates of hydrographic network density across prefectures (regions) in Xinjiang over the past 120 years (1900–2020). Overall, after experiencing fluctuations in the early 20th century, hydrographic density has shown a general increasing trend in most regions since the 1960s. However, substantial regional heterogeneity is evident, reflected in the following patterns:

1. 1900–1930s: Initial divergence among regions. Turpan exhibited the most significant increase (+ 121.4%), likely associated with the early expansion of oasis agriculture. Kizilsu (+ 46.0%) and Bayingolin (+ 31.6%) also showed notable positive growth, indicating the early development of oasis economies. In contrast, Aksu (–32.4%), Kashgar (–30.9%), and the Urumqi–Changji region (–31.5%) experienced substantial declines (Fig. 5a).
2. 1930–1960s: Strong north–south polarization. Turpan’s hydrographic density sharply decreased (–67.0%), along with marked declines in Hami (–36.4%), Bayingolin (–34.3%), and Hotan (–33.4%). Conversely, Kashgar (+ 76.1%), Urumqi–Changji (+ 49.8%), Tacheng (+ 48.8%), and Altay (+ 39.6%) recorded rapid increases (Fig. 5b).
3. 1960–1990s: Widespread expansion driven by water conservancy development. Most regions experienced rapid growth in hydrographic density. The most pronounced increases occurred in Hotan (+ 62.2%), Bayingolin (+ 43.3%), and Aksu (+ 35.4%). Kashgar (+ 27.9%) and Altay (+ 28.3%) also sustained strong growth. Only Turpan (–12.6%), Hami (–11.2%), and Bortala (–3.9%) exhibited declines, reflecting constraints imposed by local eco-hydrological conditions (Fig. 5c).
4. 1990–2020s: Slower growth and convergence across regions. Bortala (+ 37.4%) and Altay (+ 29.4%) continued to increase substantially, reflecting ongoing water development in the northern border region. Bayingolin (+ 27.7%), Turpan (+ 26.9%), and Kizilsu (+ 21.2%) maintained steady growth. In contrast, the Urumqi–Changji region (–1.9%) and Ili (–2.7%) showed slight declines (Fig. 5d).

## Discussion

### Differentiated climatic and human drivers of hydrographic network evolution

The long-term evolution of hydrographic network density (HND) in arid Xinjiang reflects the combined influences of natural hydrological regimes and intensified human modification of water systems over the past century. At the regional scale, spatial aggregation patterns of HND underwent a clear transition from early localized clustering and spatial dispersion to widespread aggregation and structural consolidation, suggesting a progressive shift toward a more organized and mature hydrographic system.

**Fig. 6:**
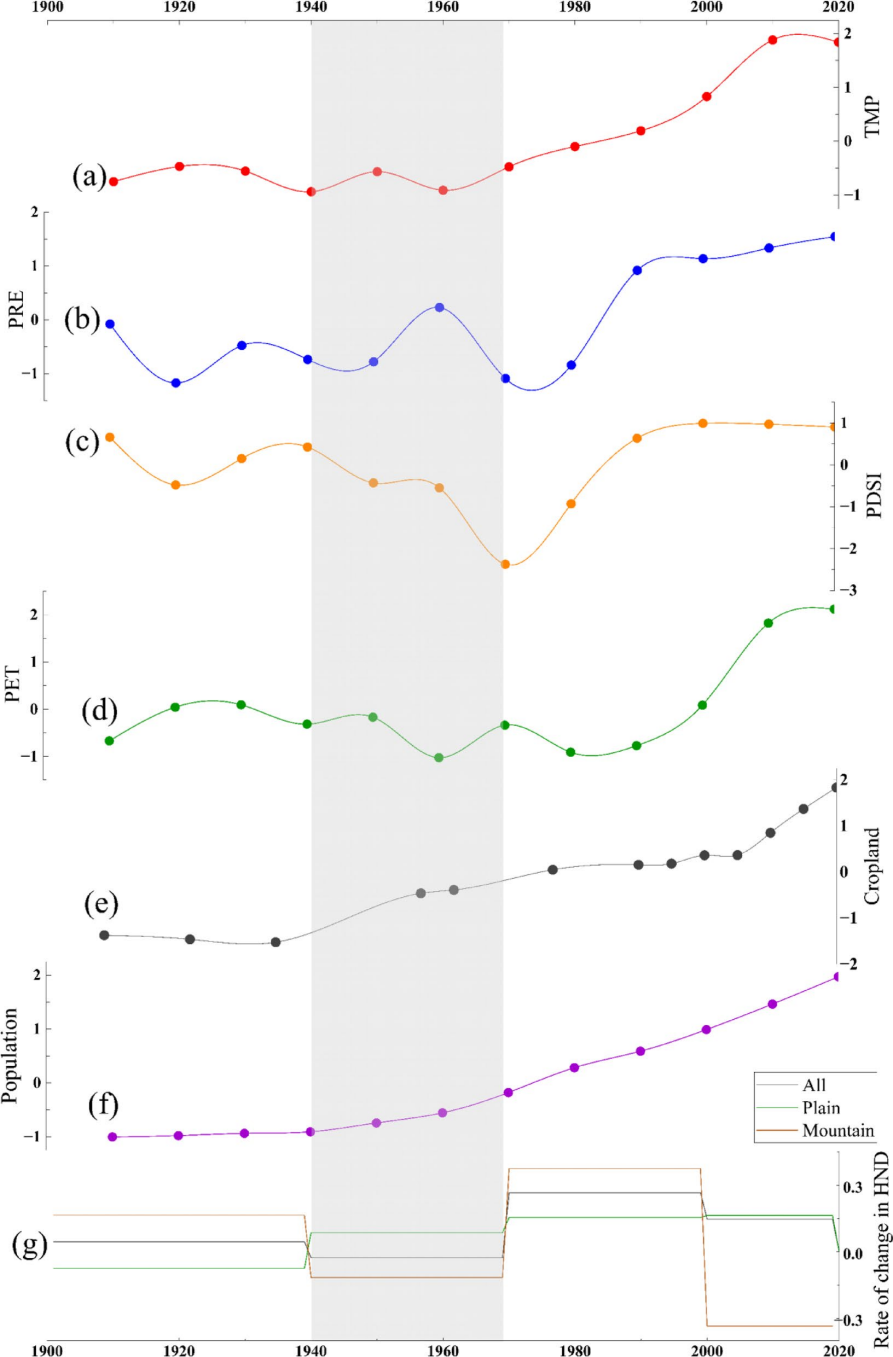
Relationship between the decadal cumulative mean of driving factors and the change rate of hydrographic network density in Xinjiang over the past 120 years. Panels (**a**–**f**) show climatic and human activity variables connected by spline curves. Mountain and oasis–plain HND values were derived from 10 km × 10 km grid-level HND data, with zone classification based on mean elevation extracted using the Zonal Statistics as Table tool (ArcGIS 10.8.2) applied to the SRTM DEM (90 m resolution); grid cells with mean elevation ≥ 1500 m were assigned to the mountainous zone and those below to the oasis-plain zone. Panel (g) shows HND change rates aggregated over four intervals: 1901–1939, 1940–1969, 1970–1999, and 2000–2019. Figure created by the authors using OriginPro 2026 (OriginLab Corporation, Northampton, MA, USA) and ArcGIS 10.8.2 (Esri Inc., Redlands, CA, USA).

Areas of persistently high HND generally correspond to regions characterized by either abundant natural water resources or well-developed irrigation infrastructure, functioning as core zones of hydrographic concentration. In mountainous regions, where artificial canal density is comparatively low, HND variations are interpreted as primarily reflecting natural hydrological responses to climatic forcing, thereby reducing the confounding influence of artificial canals on climate–HND correlations. In mountainous areas, where water supply is dominated by snow and glacier meltwater, variations in HND closely track climatic fluctuations, particularly wet–dry phases reflected by aridity-related indices (Fig. 6). In contrast, oasis–plain regions exhibit a different trajectory, with spatial patterns increasingly decoupled from short-term climatic variability and shaped by longer-term structural processes (Fig. 6).

A critical turning point in the evolution of HND occurred around the 1960s. Prior to this period, hydrographic networks–especially in mid- and lower-reach plain areas–were relatively unstable, characterized by frequent expansion and contraction as well as shifting spatial configurations. After the 1960s, high–high (HH) clusters re-emerged and expanded rapidly, signaling a transition toward a more integrated and consolidated hydrographic structure. Taken together, the evolution of HND in Xinjiang can be summarized as a long-term sequence: early localized expansion, mid-century divergence, widespread post-1960 spatial reorganization and regional expansion, and recent slowdown accompanied by regional convergence. This trajectory highlights the increasing interaction and overlaps between climatic constraints and human interventions in shaping the spatiotemporal organization of regional water systems (Fig. 6; Appendix A).

### Policy-driven restructuring of plain hydrographic systems

In contrast to mountainous regions, changes in HND across the Xinjiang plains are substantially larger in magnitude and are primarily driven by agricultural expansion and water-management policies. Traditional irrigation districts along the margins of the Tarim Basin had already developed relatively mature canal systems prior to the 20th century, resulting in hybrid hydrographic structures composed of natural river channels and artificial canals^28^. These configurations were not the outcome of natural processes alone but were shaped by long-standing land-reclamation institutions, agricultural demands, and prevailing water-governance practices.

From the late Qing to the early Republican period (approximately 1880–1920s), agricultural reclamation expanded rapidly, while social instability and warfare in the 1930s led to a sharp decline in cultivated land and a corresponding reduction in plain-area hydrographic density^27^. Following the establishment of the People’s Republic of China, irrigation development became increasingly institutionalized, accompanied by large-scale canal construction and rapid expansion of irrigated areas^20,27^. This phase corresponds closely with the spatial reorganization and overall upward trend in HND observed since the 1960s. Institutions such as the Xinjiang Production and Construction Corps played a central role in reshaping plain hydrographic systems through the construction of main canals, lateral canals, and diversion networks, substantially enhancing water-network density and spatial connectivity^18,20,29^.

Rather than representing isolated or short-term interventions, the expansion and contraction of hydrographic networks in the plains followed systematic, region-wide processes driven by irrigation development, oasis agricultural expansion, and policy-oriented water management. Since the early 21st century, national policies emphasizing ecological protection and water-saving irrigation have promoted more regulated water-resource management, contributing to a gradual stabilization in the growth rate of plain-area hydrographic networks^30^. Overall, the evolution of plain hydrographic systems in Xinjiang underscores the central role of policy regimes and engineering interventions in restructuring water networks under arid environmental conditions.

Previous studies on historical water-system changes in Xinjiang, particularly in the Tarim Basin, have consistently documented pronounced hydrological and ecological transformations since the late Qing period^7,31–34^. These studies attribute major changes to intensified irrigation, water diversion, and dam construction, which collectively reorganized river systems under combined natural and anthropogenic pressures.

The spatiotemporal patterns identified in this study are broadly consistent with these findings. Prior to the mid-20th century, mid-and lower-reach river systems exhibited high instability, characterized by frequent channel shifting and braiding. Following the 1960s, large-scale water-management projects increasingly constrained natural channel migration, leading to a more stabilized hydrographic structure^19^. This correspondence reinforces the interpretation that engineering interventions played a central role in reshaping plain-area water networks during the modern period.

### Temporal persistence, threshold shifts, and implications for arid-region water systems

**Fig. 7:**
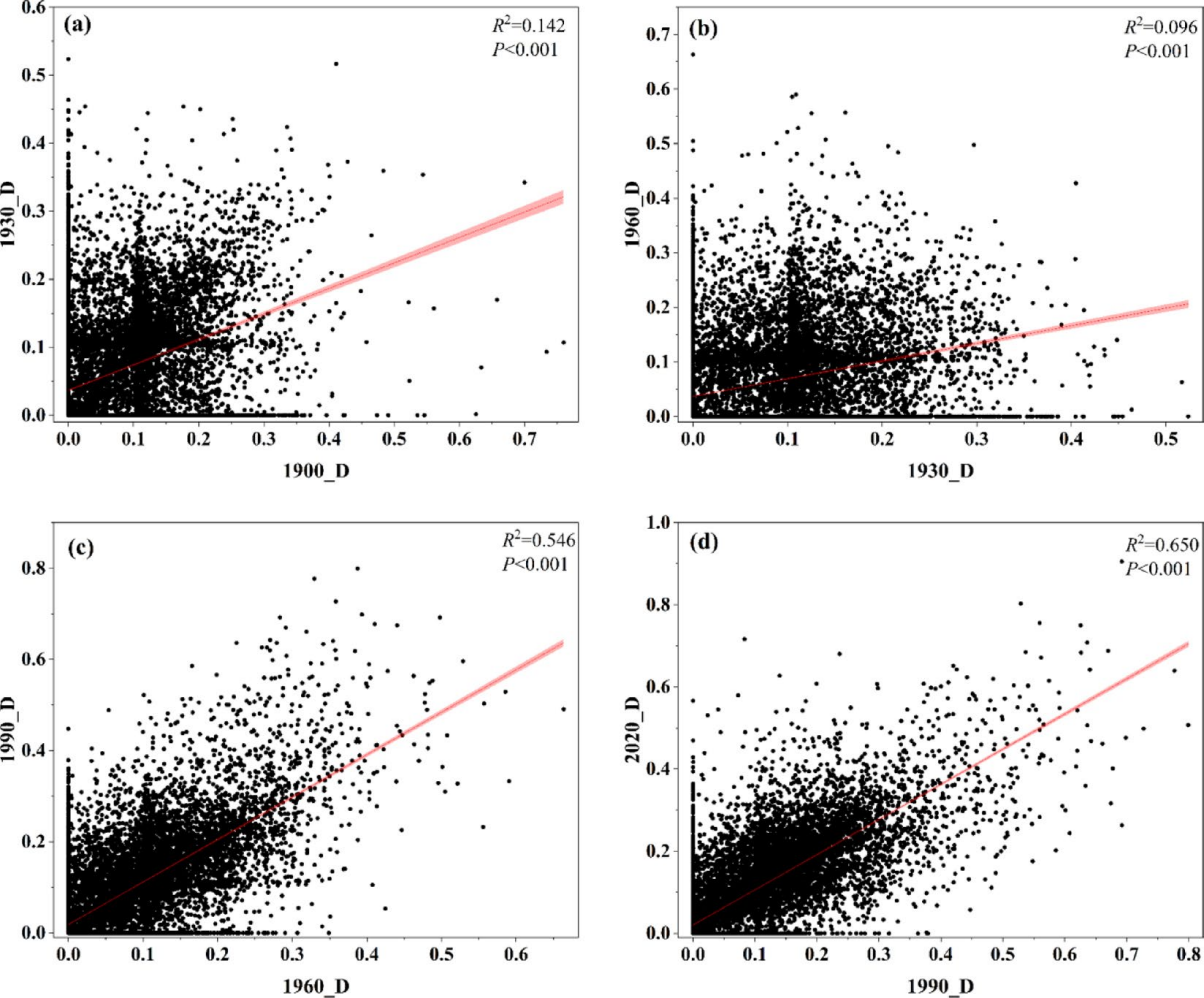
Correlations of hydrographic network density (HND) patterns across consistent time sections at a 10-km grid resolution. Panels (**a**–**d**) show HND correlation during the periods 1900–1930s, 1930–1960s, 1960–1990s, and 1990–2020s, respectively. Scatter plots and Pearson correlation coefficients were calculated and visualized using OriginPro 2026 (OriginLab Corporation, Northampton, MA, USA). Spatial data processed using ArcGIS 10.8.2 (Esri Inc., Redlands, CA, USA).

The spatial pattern of HND changed significantly during the mid-20th century, particularly around the 1960s, over the past 120 years. The period between the 1930s and the 1960s exhibits the lowest correlation coefficient compared with other time sections (*r* = 0.096, *p* < 0.01), indicating a pronounced pattern shift during this transitional phase. At the same time, significant inter-period correlations were observed among temporally adjacent sections, revealing a clear pattern persistence across consecutive periods (Fig. 7).

The Pearson correlation scatterplots show that all correlation coefficients between adjacent time sections are statistically significant (*p* < 0.001) (Fig. 7b). This result suggests that, despite long-term structural changes, HND patterns retained substantial spatial similarity between neighboring periods, reflecting short-term continuity in hydrographic network configuration.

### Methodological uncertainties and future directions

This study reconstructed a 120-year time series of HND using historical maps, but such maps inherently carry measurement uncertainties and representational limitations.

**Fig. 8:**
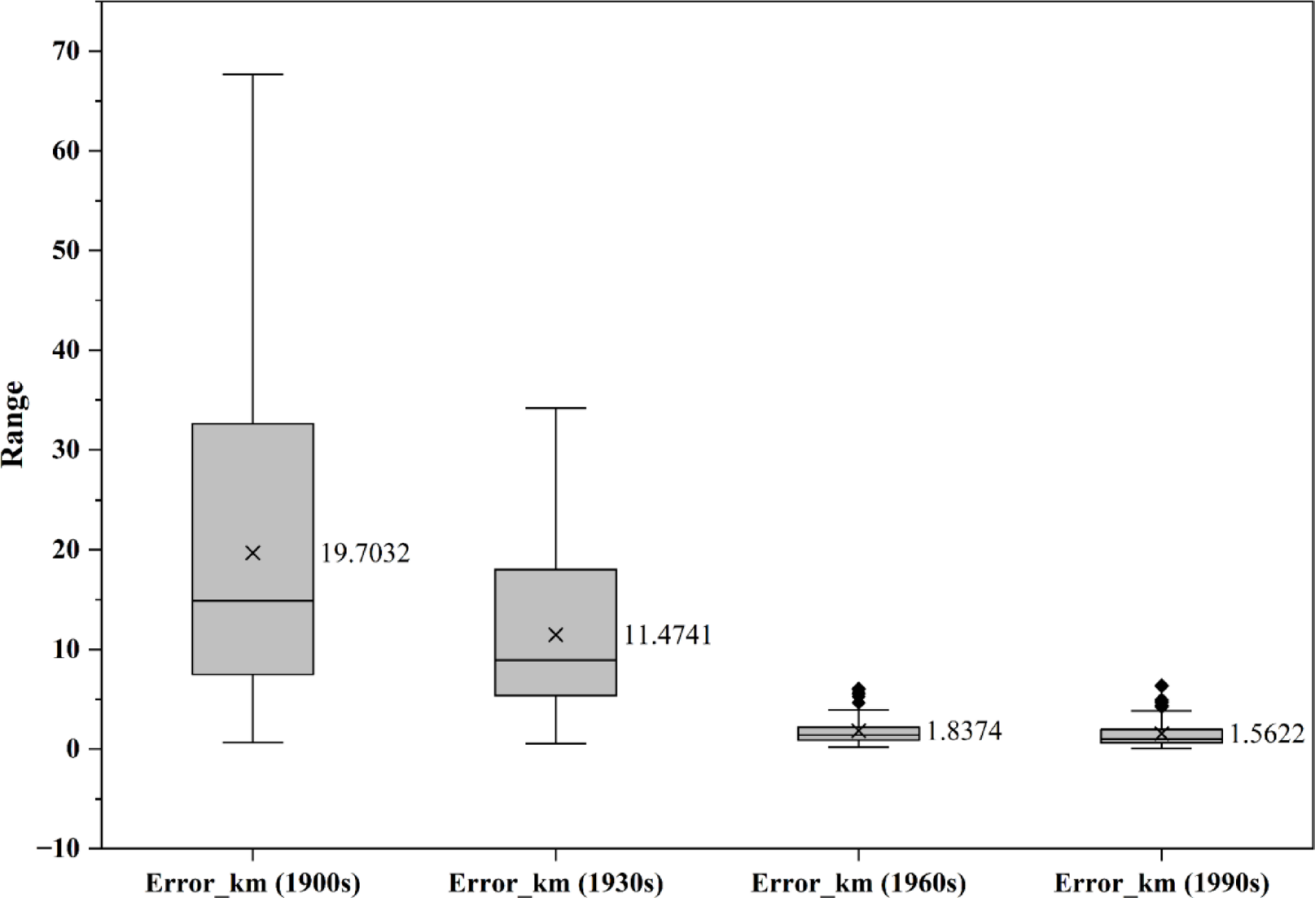
Spatial distribution of positional errors for all map sheets across different periods used to assess positional uncertainty. Spatial data processed using ArcGIS 10.8.2 (Esri Inc., Redlands, CA, USA); figure created using Origin 2026 (OriginLab Corporation, Northampton, MA, USA).

First, to evaluate spatial accuracy, we compared county-level administrative centers from the 1900s, 1930s, 1960s and 1990s (262 points total) with contemporary 1:1,000,000 standard geographic datasets (National Catalogue Service for Geographic Information, https://www.webmap.cn/commres.do?method=result100W). Positional errors were quantified using the Haversine formula (spherical Earth radius = 6,371 km), which accounts for the curvature of the Earth’s surface. Positional error decreased from an average of 19.70 km in the 1900s to 1.56 km in the 1990s, with no systematic directional bias–reflecting early surveying limitations and random distortion (Fig. 8). By using a unified Albers equal-area projection and summarizing data within consistent 10 km × 10 km grids, we effectively reduced biases caused by varying map scales, ensuring temporal comparability.

Second, aligning historical and modern hydrographic datasets poses inherent difficulties, especially in arid regions where modern datasets include many ephemeral channels. Because flow capability varies seasonally, decadally, and with human intervention, this study removed dry channels from the 2020 dataset to maintain temporal coherence. However, future work should incorporate dynamic assessment of ephemeral river systems.

Finally, the modern water-system landscape in Xinjiang interweaves natural rivers and artificial canals, yet historical maps rarely distinguish between the two. Consequently, this study cannot fully separate their spatial patterns or ecological roles. Future research should adopt small catchments as the basic unit and integrate high-resolution imagery, historical records, field surveys, and GIS methods to systematically classify and quantify natural vs. artificial channels. Understanding their distinct contributions and ecological impacts will strengthen interpretations of long-term hydrographic evolution and support water-resource management and ecological restoration.

## Conclusion

This study reconstructs the long-term evolution of hydrographic network density (HND) in arid Xinjiang from 1900 to 2020, revealing a systematic transformation of regional water systems over more than a century. The results demonstrate a clear shift from early spatial dispersion to increasing aggregation and structural stabilization, with the mid-20th century–particularly the 1960s–representing a critical turning point in hydrographic organization.

The findings highlight a differentiated response mechanism between geomorphological zones. Mountain water systems were primarily shaped by climatic variability and aridity conditions, whereas oasis–plain systems experienced profound restructuring driven by irrigation expansion, land reclamation, and policy-led water conservancy development. Over time, climatic forcing and human engineering increasingly interacted, jointly shaping the spatiotemporal configuration of the regional hydrographic network.

By integrating historical cartography with GIS-based spatial analysis at a regional scale, this study advances methodological approaches for reconstructing long-term water-system dynamics in data-scarce arid environments. Beyond the Xinjiang case, the results underscore the importance of considering both climate variability and institutional interventions when assessing historical and future water-security challenges in arid regions. This long-term perspective is essential for informing sustainable water management and adaptation strategies under continued climate change.

## Figures and Tables

**Fig. A1:**
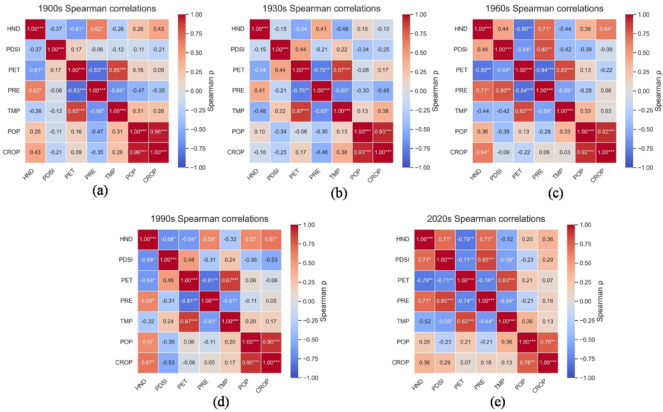
Spearman correlation heatmap between hydrographic network density (HND) and climatic and human activity factors across five representative periods over the past 120 years. Correlation analysis and heatmap visualization were performed using Python 3 with seaborn 0.13.2, matplotlib 3.10.0, scipy 1.15.3, pandas 2.2.3, and numpy 2.1.3 (Python Software Foundation, https://www.python.org).

## Data Availability

The digitized hydrographic vector features and derived 10 km grid-based hydrographic network density (HND) datasets for the five periods (1900s–2020s) are publicly available at Zenodo (https://doi.org/10.5281/zenodo.18125011).

## Appendix A

To investigate temporal variations in the correlations between HND and climatic (potential evapotranspiration (PET), precipitation (PRE), Palmer Drought Severity Index (PDSI), and mean temperature (TMP) and human activity factors (cropland area and population), we selected five representative periods (1900s, 1930s, 1960s, 1990s, and 2020s) aligned with the availability of historical records. Spearman rank correlation was adopted for all analyses, as it does not require unit consistency or distributional assumptions, making it appropriate for comparing variables with different units and scales across historical periods.

For trend visualization in Fig. 6, climatic variables were aggregated into sliding 10-year windows (1901–1910, 1911–1920, …, 2011–2020) covering the full 120-year time series. For the period-specific correlation analysis in this appendix, nine-year windows centered on each map’s reference year were used instead (1901–1909 for the 1900s, 1927–1935 for the 1930s, 1956–1964 for the 1960s, 1989–1997 for the 1990s, and 2011–2019 for the 2020s), to better align climate data with the hydrographic conditions represented in each historical map.

Population and cropland data represented snapshot estimates from contemporary sources: early 20th-century records primarily from *Xinjiang Tuzhi* (1911)^35^, supplemented by Chen (1990)^36^ for 1900s population; 1930s population estimates from Hu Huanyong’s related works^37^ and cropland data from Zhang (1969)^38^; 1960s population from the *Xinjiang Uygur Autonomous Region census compilation* (1983)^39^ and cropland from internal agricultural statistics (1980)^40^; 1990s data from *Xinjiang Statistical Yearbook* (1996)^41^; and 2020s population from the seventh national population census by-county tabulation (2022)^42^ and cropland from *Xinjiang Statistical Yearbook (2020)*^43^. As population records prior to 1949 are available only for selected years (1902, 1911, 1916, 1919, 1926, 1928, 1930, 1935, and selected years thereafter), linear interpolation between documented data points was applied to estimate values for intervening years before decadal aggregation.

All variables were aggregated across the 12 regions delineated in Fig. 5 to ensure spatial consistency across periods.

The results indicate that the dominant driving factors of HND varied substantially among different periods (Figure A1). In the 1900s, HND exhibited strong positive correlations with PRE (*r* = 0.62, *p* < 0.05) and strong negative correlations with PET (*r* = − 0.61, *p* < 0.05), suggesting a pronounced climatic control during this period. In the 1930s, no statistically significant correlations were observed between HND and the examined variables; however, PET still showed the strongest association among both natural and human-related factors (*r* = − 0.54), indicating a continued influence of aridity conditions (Figure A1).

In the 1960s, climatic and human factors jointly emerged as key drivers of HND. PET (*r* = − 0.80, *p* < 0.01), PRE (*r* = 0.71, *p* < 0.05), and cropland area (Crop) (*r* = 0.64, *p* < 0.05) became the leading correlated variables (Figure A1). In the 1990s, the PDSI (*r* = − 0.68, *p* < 0.05), cropland (*r* = 0.67, *p* < 0.05), and PET (*r* = − 0.64, *p* < 0.05) showed the strongest correlations with HND, highlighting the combined effects of climatic aridity and agricultural expansion (Figure A1 and Fig. 6). In the 2020s, PET (*r* = − 0.79, *p* < 0.01), PRE (*r* = 0.71, *p* < 0.05), and PDSI (*r* = 0.71, *p* < 0.05) became the most influential factors associated with HND.
